# Creatine supplementation and resistance training: a comparison between novice and experienced lifters - a systematic review and dose-response meta-analysis

**DOI:** 10.1080/15502783.2025.2586523

**Published:** 2025-12-23

**Authors:** Damoon Ashtary-Larky, Shooka Mohammadi, Leila Hajizadeh, Seyed Amir Hossein Mousavi, Scott C. Forbes, Darren G. Candow, Jose Antonio

**Affiliations:** a Nutrition and Metabolic Diseases Research Center, Ahvaz Jundishapur University of Medical Sciences, Ahvaz, Iran; b Department of Social and Preventive Medicine, Faculty of Medicine, University of Malaya, Kuala Lumpur, Malaysia; c Department of Sport Physiology, Marvdasht Branch, Islamic Azad University, Marvdasht, Iran; d Student Research Committee, Ahvaz Jundishapur University of Medical Sciences, Ahvaz, Iran; e Department of Physical Education Studies, Faculty of Education, Brandon University, Brandon, MB, Canada; f Faculty of Kinesiology and Health Studies, University of Regina, Regina, SK, Canada; g Department of Health and Human Performance, Nova Southeastern University, Davie, FL, USA

**Keywords:** Creatine, body composition, fat-free mass, muscle hypertrophy, resistance training

## Abstract

**Background:**

Creatine (Cr) supplementation is well established for enhancing fat-free mass (FFM) when combined with resistance training (RT). However, the influence of prior training experience on supplementation efficacy remains unknown.

**Objective:**

This systematic review and dose–response meta-analysis of controlled trials evaluated the effects of Cr supplementation combined with RT on body composition, with particular emphasis on the differences between trained (experienced) and untrained (novice) individuals.

**Methods:**

A systematic search of major databases was conducted to identify controlled trials published until March 2025. The effects of Cr supplementation on body mass, body mass index (BMI), FFM, fat mass (FM), and body fat percentage (BFP) were examined using random-effects meta-analysis.

**Results:**

A pooled analysis of 61 trials revealed that Cr supplementation significantly increased FFM (weighted mean difference [WMD]: 1.39 kg; 95% confidence intereval (CI): 1.07,1.70; *p* < 0.001) and body mass (WMD: 0.89 kg; 95% CI: 0.76,1.01; *p* < 0.001) without significant effects on FM, BMI, and BFP. Trained individuals exhibited greater, though non-significant, gains in FFM (1.82 vs. 1.23 kg) compared with untrained participants, despite similar increases in total body mass. Dose–response analyses identified significant relationships between Cr dose and changes in body mass and BMI. Furthermore, supplementation duration was associated with changes in BFP and body mass.

**Conclusion:**

Both novice and experienced lifters gained FFM with Cr supplementation compared to placebo. The increase in FFM was approximately 0.6 kg (≈50%) greater in experienced participants; however, this between-group difference was not statistically significant.

## 1. Introduction

Creatine (Cr) is among the most extensively investigated and effective ergogenic aids [1]. In contrast to several other ergogenic aids [2–4], Cr supplementation has been demonstrated to improve body composition [5]. When combined with resistance training (RT), Cr supplementation consistently results in significantly greater increases in fat-free mass (FFM) compared to training alone [6–8]. Meta-analyses have quantified these benefits, indicating that individuals who supplement with Cr during a structured training program gain, on average, 1–2 kg more lean tissue than those receiving a placebo [5,7]. Further, some investigations have reported small, yet statistically significant decreases in body fat percentage (BFP) following Cr supplementation [5].

The beneficial effects of Cr supplementation on body composition have been documented across a wide range of populations, from untrained (novice) individuals to highly trained (experienced) athletes [9]. In previously untrained individuals, the addition of Cr to a newly initiated RT program has been shown to significantly enhance adaptations. For instance, novice lifters supplementing with Cr have demonstrated approximately 20–25% greater strength gains over several weeks of training compared to placebo, along with significant increases in muscle hypertrophy [10]. It has been reported that men and women with no prior RT experience exhibited greater gains in muscle thickness and leg press strength after six weeks of training with Cr supplementation compared to training alone [11]. These findings suggest that novice trainees, who typically experience rapid neuromuscular adaptations in the early stages of RT, can further augment their initial strength and hypertrophy gains through Cr supplementation.

Cr supplementation has also been shown to elicit positive adaptations in experienced resistance-trained individuals, including athletes and strength-trained adults [12]. While well-trained individuals typically experience smaller incremental improvements due to their proximity to genetic or training plateaus, numerous studies indicate that Cr can enhance these gains. Research conducted on trained populations, such as collegiate athletes, bodybuilders, and powerlifters, suggests that Cr supplementation can further augment muscle hypertrophy and performance outcomes. For instance, in an 8-week trial involving resistance-trained men, those supplementing with Cr exhibited a significant increase in FFM, along with a reduction in lower-limb fat mass (FM) compared to the placebo group [13]. These findings align with the broader body of literature, which demonstrates that even in highly trained individuals, Cr supplementation can facilitate additional muscle accretion and improvements in body composition.

The extent to which prior training status modulates the magnitude of Cr-induced adaptations remains an open question, particularly in individuals engaged in RT. Some evidence suggests that the relative benefits of Cr supplementation are independent of training history. Notably, a meta-analysis conducted over two decades ago synthesized findings from multiple Cr studies and reported no significant differences in body composition or performance effect sizes between trained and untrained individuals, suggesting comparable ergogenic benefits across both groups [14]. However, it is important to note that this meta-analysis did not specifically assess differences in Cr responsiveness among resistance-trained individuals. Evidence suggests that previously untrained individuals engaging in RT may experience greater relative adaptations from Cr supplementation compared to those with prior training experience [15]. Nevertheless, while the existing literature lacks sufficient direct comparisons between these populations, some evidence suggests that protein supplementation may be more effective in trained individuals than in untrained ones [16], highlighting the need for further investigation.

Given the widespread use of Cr supplementation among novice and experienced resistance-trained individuals, understanding the influence of training status on its efficacy is of both practical and theoretical significance. To date, a critical gap remains in the literature: no prior meta-analysis has systematically investigated how an individual's RT history moderates the body composition outcomes associated with Cr supplementation. Existing reviews and meta-analyses have generally pooled heterogeneous populations or restricted analyses to narrow demographic groups, thereby overlooking the moderating role of training experience [5,17–21]. Consequently, a systematic investigation is necessary to address these uncertainties. By synthesizing data from studies that included both trained and untrained, or novice and experienced, populations, this meta-analysis aims to enhance statistical power and detect potential interactions between training status and Cr’s effects that may not be evident in individual studies. Therefore, the objective of this meta-analysis is to quantitatively assess the impact of prior RT experience on the efficacy of Cr supplementation in modifying body composition.

## 2. Methods

The protocol for this systematic review and meta-analysis was registered with the International Prospective Register of Systematic Reviews (PROSPERO; registration ID: CRD420251034695). This study adhered to the Preferred Reporting Items for Systematic Reviews and Meta-Analyses (PRISMA) guidelines [22].

### 2.1. Search strategy

A comprehensive literature search was conducted in PubMed/MEDLINE, Scopus, and Web of Science from database inception to March 2025 with no restrictions on language or publication date. Two investigators independently screened all retrieved records to identify eligible controlled trials with parallel or crossover designs. The search strategy was structured around four main trial components: population (adults), intervention (Cr supplementation combined with RT), comparator (placebo or no supplementation with RT), and outcomes (body mass, body mass index [BMI], FM, BFP, and FFM). The search terms were as follows: (Creatine) AND (“body mass” OR “body mass index” OR “weight loss” OR “obesity” OR “BMI” OR “weight reduction” OR “abdominal obesity” OR “central obesity” OR “visceral obesity” OR “obese” OR “overweight” OR “fat mass” OR “body fat” OR “FM” OR “body fat percentage” OR “BFP” OR “fat-free mass” OR “FFM”).

### 2.2. Eligibility criteria

EndNote software was used to manage the references. Two investigators independently reviewed the titles, abstracts, and full texts. A third investigator resolved disagreements. Eligible studies were selected based on the PICOS framework. Participants (*P*) included adults aged 18 years or older, of any sex, who engaged in RT. Prior training experience was not an inclusion criterion; however, for analytical purposes, studies were later categorized according to participants’ baseline training status (trained or untrained). Studies involving pregnant women or participants under 18 years of age were excluded. Intervention (I) consisted of Cr supplementation combined with a structured RT program. To isolate the effects of Cr, studies that used Cr in combination with other performance-enhancing or multi-ingredient supplements were excluded. Comparison (C) groups were required to include a placebo or non-intervention control condition. Outcomes (O) included changes in body composition parameters such as body mass, body mass index (BMI), FM, FFM, or BFP, and studies were required to report baseline and post-intervention quantitative data for at least one of these outcomes. Study design (S) was limited to controlled trials (randomized or non-randomized) published in peer-reviewed journals, while observational studies, uncontrolled trials, conference abstracts, and non–peer-reviewed reports were excluded.

### 2.3. Data extraction

Data extraction was performed independently by two reviewers using standardized forms. The extracted data included study characteristics (first author name, publication year, design, setting, and sample size), participant demographics (age, sex, and baseline training status), RT intervention details (duration and frequency), and Cr supplementation protocols (dose, duration, and loading regimen). The outcomes included baseline and post-intervention values for body mass, BMI, FM, BFP, and FFM measurements. Any discrepancies were resolved through discussion or consultation with a third reviewer.

### 2.4. Risk of bias assessment

The methodological quality of the included trials was assessed independently by two investigators using the Cochrane Risk of Bias 2 (RoB 2) tool [23]. This tool was used to assess potential sources of bias, including selection, performance, detection, attrition, and reporting biases. Each domain was classified as presenting a low, high, or unclear risk of bias [23].

### 2.5. Statistical analysis

Meta-analyses were conducted using Stata software (version 17). Trial outcomes were reported as mean ± standard deviation (SD) [24]. Effect sizes were calculated as weighted mean differences (WMDs) with 95% confidence intervals (CIs). A random-effects model was applied to estimate the pooled WMDs [25], and heterogeneity was assessed using Cochran’s Q test and the I² statistic [26]. I² values of 25%, 50%, and 75% were considered to represent low, moderate, and high heterogeneity, respectively [27].

Subgroup analyses were performed to investigate the potential sources of this heterogeneity. Trials were stratified by study duration (≤30 vs. >30 days), type of Cr (creatine monohydrate (CrM) vs. other forms), daily supplementation dose (≤5 vs. >5 g/day), and total supplementation dose (≤300 vs. >300 g). Additional subgroup analyses were conducted according to participant characteristics, including baseline BMI (normal, overweight, and obese), sex (both sexes, men, and women), and age (≤40 vs. >40 years old). Supplementation protocols (loading only, maintenance, loading with short-term maintenance, loading with long-term maintenance, or high-dose maintenance) and baseline training status (trained vs. untrained) were also evaluated. For between-group comparisons between trained and untrained individuals, Cohen’s *d* with the corresponding 95% CIs was calculated. Cohen’s d was interpreted as small (0.2), medium (0.5), and large (0.8), reflecting the magnitude of differences between groups.

Sensitivity analyses were conducted by sequentially excluding individual trials to assess the robustness of the study's findings. Publication bias was assessed using funnel plots in combination with Begg’s [28] and Egger’s tests [29]. Moreover, a fractional polynomial model was applied to identify potential nonlinear dose–response relationships between trial duration or Cr supplementation dose and changes in outcomes. Meta-regression analyses were also performed to examine the potential linear associations between Cr dosage or trial duration and outcome variations [30]. Statistical significance was set at *P* < 0.05.

## 3. Results

### 3.1. Study selection

**Figure 1.**
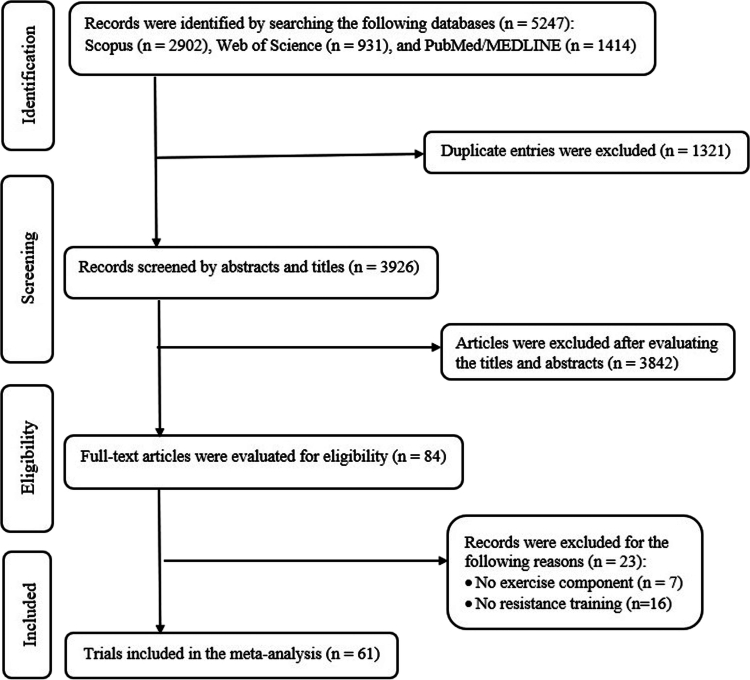
Flow diagram of study selection.

As illustrated in Figure 1, an extensive systematic search was initially performed across online databases, yielding a total of 5,247 studies. Of these, 1,321 were identified as duplicates and subsequently removed, while 3,842 irrelevant studies were excluded after a thorough screening of titles and abstracts. After a comprehensive full-text evaluation, 23 studies were excluded due to insufficient extractable data or ineligible interventions, specifically: no exercise component [31–37], exercise limited to non-resistance modalities e.g. aerobic [38–43] or combat sports [44–47], or concurrent training protocols combining resistance and aerobic exercise [48–53]. Ultimately, 61 controlled trials met the inclusion criteria and were included in the analysis [11,13,54–112].

Sixty-one trials involved 1,457 participants, including 750 individuals in the intervention group and 697 in the control group. The studies were published between 1997 and 2024, with intervention durations ranging from four to 364 days. The sample sizes varied from 8 to 38 participants. Of these trials, 56 employed a parallel-group design [11,13,54–59,61–81,83–90,92–94,96–111], while five utilized a crossover design [60,82,91,95,112]. The studies were conducted in the United States of America (USA) [13,58–61,68,74,78,79,81,82,87,89,93,94,96–98,100,101,103–106,108,109], France [111], the United Kingdom (UK) [86,112], Canada [11,62–66,75,83,92], Australia [69,70,85], Brazil [55,56,71,77,90], Iran [57,73,99], Finland [54], New Zealand [67], Belgium [76,80,91,102], Poland [84], and Serbia [110]. Regarding participant characteristics, 10 studies included only women [38,54,65,75,77,78,89,90,102,107,110] focused exclusively on men [13,55–58,60,61,64,66,68–71,73,74,76,81,82,84–88,91,93,94,96,98–101,103–106,108,109,112], and 13 involved both sexes [11,59,62,63,67,72,79,80,83,92,95,97,111]. The majority of trials (*n* = 56) were double-blind [11,54–72,74–84,86–105,107–109,111,112], typically blinding both participants and investigators,while five trials [60,82,91,95,112] did not implement blinding. The detailed characteristics of the included studies are presented in Table 1.

**Table 1.** Characteristics of included studies in meta-analysis.

Reference	Study region	Study design	Participants	Sex	Sample size		Trial duration (days)	Means age		Intervention				
					IG	CG		IG	CG	Loading	Cr type	Supplantation protocols	Exercise	CG
[102]	Belgium	R, P, PC, DB	Healthy, sedentary non-vegetarian females	♀	10	9	75	19−22	19−22	L + LM	CrM	20 g/d × 4 d + 5 g/d × 71 d	RT	PL (MD)
[111]	France	R, P, PC, DB	Elderly individuals	♂/♀	8	8	52	71	69.3	L + LM	CrM	20 g/d × 5 d + 3 g/d × 47 d	RT	PL (Glucose)
[87]	USA	R, P, PC, DB	Football players	♂	11	14	28	18−23	18−23	HDM	CrM	15.75 g/d × 28 d	RT	PL (Phosphagen HP)
[112]	UK	R, C, PC, DB	Healthy men	♂	5	5	5	28	28	JL	CrM	10 g/d × 5 d	RT	PL (Glucose)
[85]	Australia	R, P, PC	Male powerlifters	♂	9	9	26	25.5	28.1	L + SM	CrM	20 g/d × 5 d + 5 g/d × 21 d	RT	PL (Glucose)
[105]	USA	R, P, PC, DB	Active males	♂	12	12	21	23.3	21.3	L + SM	CrM	20 g/d × 5 d + 10 g/d × 16 d	RT	PL (CHO)
[105]	USA	R, P, PC, DB	Active males	♂	12	12	21	21.9	22.3	L + SM	CrM	20 g/d × 5 d + 10 g/d × 16 d	RT	PL (CHO)
[76]	Belgium	R, P, PC, DB	Healthy untrained males	♂	8	10	42	22	22	L + LM	CrM	21 g/d × 5 d + 3 g/d × 37 d	RT	PL (MD)
[94]	USA	R, P, PC, DB	Resistance-trained males	♂	11	7	42	19−29	19−29	L + LM	CrM	20 g/d × 3 d + 10 g/d × 39 d	RT	PL (MD)
[94]	USA	R, P, PC, DB	Resistance-trained males	♂	9	7	42	19−29	19−29	L + LM	CrP	20 g/d × 3 d + 10 g/d × 39 d	RT	PL (MD)
[93]	USA	R, P, PC, DB	Male football players	♂	8	7	70	20.7	20.7	M	CrM	5 g/d × 70 d	RT	PL
[89]	USA	R, P, PC, DB	Trained females	♀	7	6	91	19.3	19	L + LM	CrM	15 g/d × 7 d + 5 g/d × 84 d	RT	PL (PowerAde)
[78]	USA	R, P, PC, DB	Resistance-trained female	♀	11	13	7	22.5	23.9	JL	CrM	25 g/d × 7 d	RT	PL (DX)
[103]	USA	R, P, PC, DB	Healthy resistance-trained men	♂	10	9	84	25.6	25.4	L + LM	CrM	25 g/d × 7 d + 5 g/d × 77 d	RT	PL (Cellulose)
[61]	USA	R, P, PC, DB	Male volunteers	♂	10	13	42	21.5	21.5	L + LM	CrM	20 g/d × 5 d + 2 g/d × 37 d	RT	PL (Sucrose)
[91]	Belgium	R, C, PC, DB	Healthy young males	♂	11	11	5	20.7	20.7	JL	CrM	20 g/d × 5 d	RT	PL (MD)
[96]	USA	R, P, PC, DB	Resistance trainers	♂	8	8	4	20.5	21.6	JL	CrM	20 g/d × 4 d	RT	PL (Sucrose)
[66]	Canada	R, P, PC, DB	Moderately active men	♂	16	14	84	70.4	71.1	L + LM	CrM	26.4 g/d × 5 d + 6.2 g/d × 79 d	RT	PL(CHO)
[109]	USA	R, P, PC, DB	Untrained males	♂	8	8	84	20.4	20.4	M	CrM	6 g/d × 84 d	RT	PL (DX)
[80]	Belgium	R, P, PC, DB	Healthy students	♂/♀	11	11	84	20−23	20−23	L + LM	CrM	20 g/d × 14 d + 15 g/d × 21 d + 5 g/d × 35 d	RT	PL (MD)
[58]	USA	R, P, PC, DB	Resistance-untrained males	♂	10	10	28	20	20	L + SM	CrM	20 g/d × 5 d + 10 g/d × 23 d	RT	PL (DX)
[84]	Poland	R, P, PC, DB	Healthy male	♂	11	10	3	21.4	20.4	L + LM	CrM	20 g/d × 5 d + 10 g/d × 14 d	RT	PL (Glucose)
[84]	Poland	R, P, PC, DB	Healthy male	♂	10	9	3	21.2	20.8	L + LM	CrM	20 g/d × 5 d + 10 g/d × 14 d	RT	PL (Glucose)
[108]	USA	R, P, PC, DB	Well-trained male	♂	8	9	70	18.8	19.2	M	CrM	3 g/d × 70 d	RT	PL (DX)
[108]	USA	R, P, PC, DB	Well-trained male	♂	8	9	70	18.8	19.2	L + LM	CrM	20 g/d × 7 d + 5 g/d × 63 d	RT	PL (DX)
[86]	UK	R, P, PC, DB	Resistance-trained men	♂	21	11	5	24.5	24.5	JL	CrM	20 g/d × 5 d	RT	PL (Glucose)
[82]	USA	R, C, PC, DB	Recreationally active male	♂	5	5	21	24	24	L + SM	CrM	20 g/d × 4 d + 2 g/d × 17 d	RT	PL (DX)
[88]	USA	R, P, PC, DB	Active males	♂	9	8	28	22.9	22.9	HDM	CrM	30 g/d × 14 d + 15 g/d × 14 d	RT	PL (MD + rice bran + sucrose)
[106]	USA	P, PC, CO	Healthy strength-trained males	♂	12	12	7	NR	NR	JL	CrM	21 g/d × 7 d	RT	NI
[62]	Canada	R, P, PC, DB	Healthy men	♂	8	7	98	68.7	68.3	M	CrM	5 g/d × 98 d	RT	PL (DX)
[62]	Canada	R, P, PC, DB	Healthy women	♀	6	7	98	70.8	69.9	M	CrM	5 g/d × 98 d	RT	PL (DX)
[104]	USA	R, P, PC, DB	Healthy resistance-trained men	♂	9	8	28	20.7	21.3	L + SM	CrM	26 g/d × 7 d + 4.33 g/d × 21 d	RT	PL (Cellulose)
[60]	USA	R, C, PC, DB	Recreationally active college-aged men	♂	5	5	21	18−40	18−40	L + SM	CrM	20 g/d × 4 d + 2 g/d × 17 d	RT	PL (MD)
[75]	Canada	R, P, PC, DB	Recreationally strength-trained women	♀	13	13	70	24.6	24.6	L + LM	CrM	19.71 g/d × 7 d + 1.97 g/d × 63 d	RT	PL
[97]	USA	R, P, PC, DB	Older adults	♂/♀	15	15	84	55−84	55−84	M	CrM	3 g/d × 84 d	RT	PL (MD)
[81]	USA	R, P, PC, DB	Male football player	♂	11	11	70	18−23	18−23	M	CrM	10.5 g/d × 70 d	RT	PL (DX)
[79]	USA	R, P, PC, DB	Individuals with PD	♂/♀	10	10	84	62.8	62.2	L + LM	CrM	20 g/d × 5 d + 5 g/d × 79 d	RT	PL (Lactose monohydrate)
[69]	Australia	R, P, PC, DB	Recreational male bodybuilders	♂	10	11	70	26	26	M	CrM	8.9 g/d × 70 d	RT	PL (PRO—CHO)
[70]	Australia	R, P, PC, DB	Recreational male bodybuilders	♂	8	7	77	25	24	L + LM	CrM	25 g/d × 7 d + 8.4 g/d × 70 d	RT	PL (CHO)
[70]	Australia	R, P, PC, DB	Recreational male bodybuilders	♂	6	5	77	25	24	L + LM	CrM	25 g/d × 7 d + 8.4 g/d × 70 d	RT	PL (WP)
[71]	Brazil	R, P, PC, DB	Male college students	♂	9	9	42	25	23	L + LM	CrM	30 g/d × 7 d + 5 g/d × 35 d	RT	PL (MD)
[74]	USA	R, P, PC, DB	Middle-aged men	♂	10	10	98	48−72	48−72	M	CrM	2.14 g/d × 98 d	RT	Sport drink
[74]	USA	R, P, PC, DB	Middle-aged men	♂	11	11	98	48−72	48−72	M	CrM	2.14 g/d × 98 d	RT	WP + Sport drink
[98]	USA	R, P, PC, DB	HIV-positive individuals	♂	19	19	98	44	44	L + LM	CrM	20 g/d × 5 d + 4.8 g/d × 93 d	RT	PL
[100]	USA	R, P, PC, DB	Non-resistance-trained men	♂	10	10	48	20.36	20.16	L + LM	CrM	20 g/d × 5 d + 5 g/d × 42 d	RT	PL (MD)
[100]	USA	R, P, PC, DB	Non-resistance-trained men	♂	10	10	48	20.83	20.16	L + LM	CrEE	20 g/d × 5 d + 5 g/d × 42 d	RT	PL (MD)
[99]	Iran	R, P, PC, DB	Healthy non-resistance-trained young men	♂	8	8	56	23.85	22.28	L + LM	CrM	23.3 g/d × 7 d + 3.88 g/d × 49 d	RT	Cellulose
[90]	Brazil	R, P, PC, DB	Postmenopausal women with knee osteoarthritis	♀	13	11	84	58	56	L + LM	CrM	20 g/d × 5 d + 5 g/d × 79 d	RT	PL (DX)
[101]	USA	R, P, PC, DB	Resistance-trained men	♂	14	15	56	21	19.8	M	CrM	5 g/d × 56 d	RT	PL (DX)
[54]	Finland	R, P, PC, DB	Non-athletic healthy women	♀	9	9	84	64	65	M	CrM	5 g/d × 84 d	RT	PL (MD)
[77]	Brazil	R, P, PC, DB	Postmenopausal women with osteopenia or osteoporosis	♀	15	15	166	67.1	63.6	L + LM	CrM	20 g/d × 5 d + 5 g/d × 161 d	RT	PL (DX)
[68]	USA	R, P, PC, DB	Healthy males	♂	10	10	84	61.4	60.7	L + LM	CrM	20 g/d × 7 d + 8.8 g/d × 77 d	RT	PL (CHO)
[65]	Canada	R,P, PC, DB	Postmenopausal women	♀	15	18	364	57	57	M	CrM	6.9 g/d × 364 d	RT	PL(MD)
[11]	Canada	R, P, PC, DB	Healthy older adults	♂/♀	15	12	224	53	57	M	CrM	7.7 g/d × 224 d	RT	PL (MD)
[11]	Canada	R, P, PC, DB	Healthy older adults	♂/♀	12	12	224	55	57	M	CrM	8.7 g/d × 224 d	RT	PL (MD)
[95]	Brazil	R, C, PC, DB	Healthy elderly	♂/♀	13	14	84	67.4	67.1	M	CrM	5 g/d × 84 d	RT	PL (MD)
[67]	New Zealand	R,P, PC, DB	Postmenopausal women	♂/♀	9	7	98	70	69	M	CrM	5 g/d × 98 d	RT	PL (MD + WP)
[83]	Canada	R, P, PC, DB	Untrained aging adults	♂/♀	14	17	84	58	57.6	M	CrM	7.83 g/d × 84 d	RT	PL (MD)
[107]	USA	R, P, PC, DB	Resistance-trained women	♀	8	9	56	22	20	M	CrM	5 g/d × 56 d	RT	PL (WP)
[57]	Iran	R, P, PC, DB	Underweight non-athlete men	♂	8	8	42	20.87	20.37	L + LM	CrEE	20 g/d × 5 d + 5 g/d × 37 d	RT	PL (Rice flour)
[55]	Brazil	R, P, PC, DB	Resistance-trained males	♂	9	9	7	22.7	24.2	JL	CrM	24.2 g/d × 7 d	RT	PL (DX)
[64]	Canada	R, P, PC, DB	Older males	♂	18	20	364	58	56	M	CrM	9.3 g/d × 364 d	RT	PL (MD)
[13]	USA	R, P, SB, CO	Resistance-trained men	♂	8	8	56	26.6	26.6	M	CrM	7.6 g/d × 56 d	RT	NI
[92]	Canada	R, P, PC, DB	Trained young adults	♂/♀	5	5	42	22	23	M	CrM	7.6 g/d × 42 d	RT	PL (Cellulose)
[92]	Canada	R, P, PC, DB	Trained young adults	♂/♀	5	5	42	22	19	M	CrM	7.4 g/d × 42 d	RT	PL (Cellulose)
[56]	Brazil	R, P, PC, DB	Healthy male individuals	♂	17	17	28	23.1	23.8	L + SM	CrM	21.9 g/d × 7 d + 2.1 g/d × 21 d	RT	PL (DX)
[59]	USA	R, P, PC, DB	Healthy young adults	♂/♀	8	10	14	28.5	30.5	M	CrM	5 g/d × 14 d	RT	PL (MD)
[63]	Canada	R, P, PC, DB	Stroke survivors	♂/♀	5	3	70	51	69	L + LM	CrM	25.1 g/d × 7 d + 8.5 g/d × 63 d	RT	PL (MD)
[72]	USA	R, P, PC, DB	Male and female collegiate athletes	♂/♀	12	6	42	19.8	19.8	M	CrM	5 g/d × 42 d	RT	PL (MD)
[72]	USA	R, P, PC, DB	Male and female collegiate athletes	♂/♀	11	5	72	19.8	19.8	M	CrM	5 g/d × 42 d	RT	PL (MD)
[73]	Iran	R, P, PC	Male soldiers	♂	12	6	42	25.3	24	M	CrHCL	2.1 g/d × 42 d	RT	PL (MD)
[73]	Iran	R, P, PC	Male soldiers	♂	12	6	42	23.8	24	M	CrM	2.1 g/d × 42 d	RT	PL (MD)
[110]	Serbia	R, P, PC	Junior women wrestlers	♀	6	6	42	18.8	18.9	L + LM	CrM	25 g/d × 7 d + 10 g on training days	RT	PL

**Abbreviations:** P, parallel; C, crossover; IG, intervention group; CG, control group; DB, double-blinded; SB, single-blinded; PC, placebo-controlled; CO, controlled; R, randomized; NR, not reported; PL, placebo; PD, Parkinson's disease; USA, United States of America; ♀, female; ♂, male; CrM, creatine monohydrate; MD, maltodextrin; RT, resistance training; CrP, creatine phosphate; CrEE, creatine ethyl ester; CrHCL, creatine hydrochloride;HIV, human immunodeficiency virus*;* DX, dextrose Phosphagen HP, Phosphagen High Performance; WP, whey protein; CHO, carbohydrate; NI, no intervention; PRO-CHO, protein–carbohydrate; L+LM, loading + long maintenance; L+SM, loading + short maintenance; HDM, high-dose maintenance; JL, just loading; M, maintenance.

### 3.2. Effect of Cr supplementation on body composition

#### 3.2.1. Body mass

The meta-analysis of 60 effect sizes from 52 studies [13,54–64,66,68–72,74–76,78–83,86–91,93–97,99–112] revealed a significant increase in body mass following Cr supplementation (WMD: 0.89 kg; 95% CI: 0.76,1.01; *p* < 0.001) (Figure 2A). Subgroup analyses indicated that the observed effects on body mass were significant among male participants, individuals aged ≤ 40 years, and across subgroups defined by trial duration, supplementation dose, total Cr intake, loading protocol, type of Cr, normal and overweight BMI, and baseline training status (Table 2).

Figure 2.Forest plots showing the weighted mean differences (WMDs) with 95% confidence intervals (CIs) for the effects of creatine (CR) supplementation on: (A) body weight (kg), (B) body mass index (BMI, kg/m²), (C) fat mass (FM, kg), (D) body fat percentage (BFP, %), and (E) fat-free mass (FFM, kg).

**Figure:**
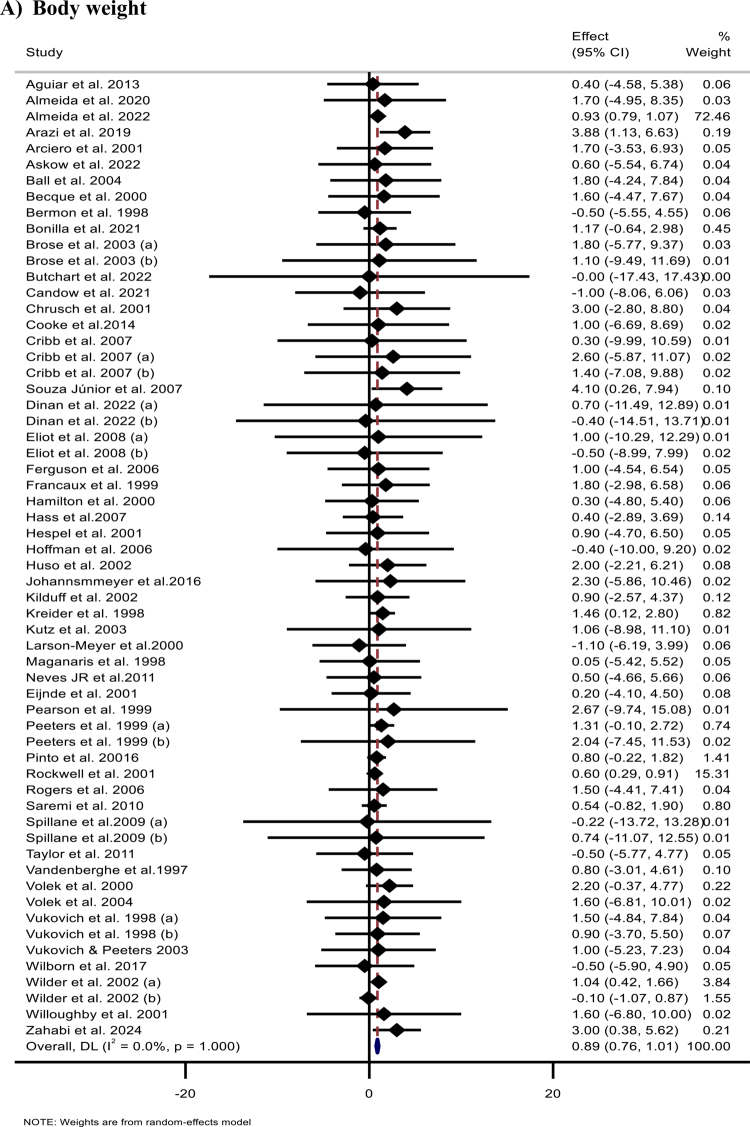

**Figure:**
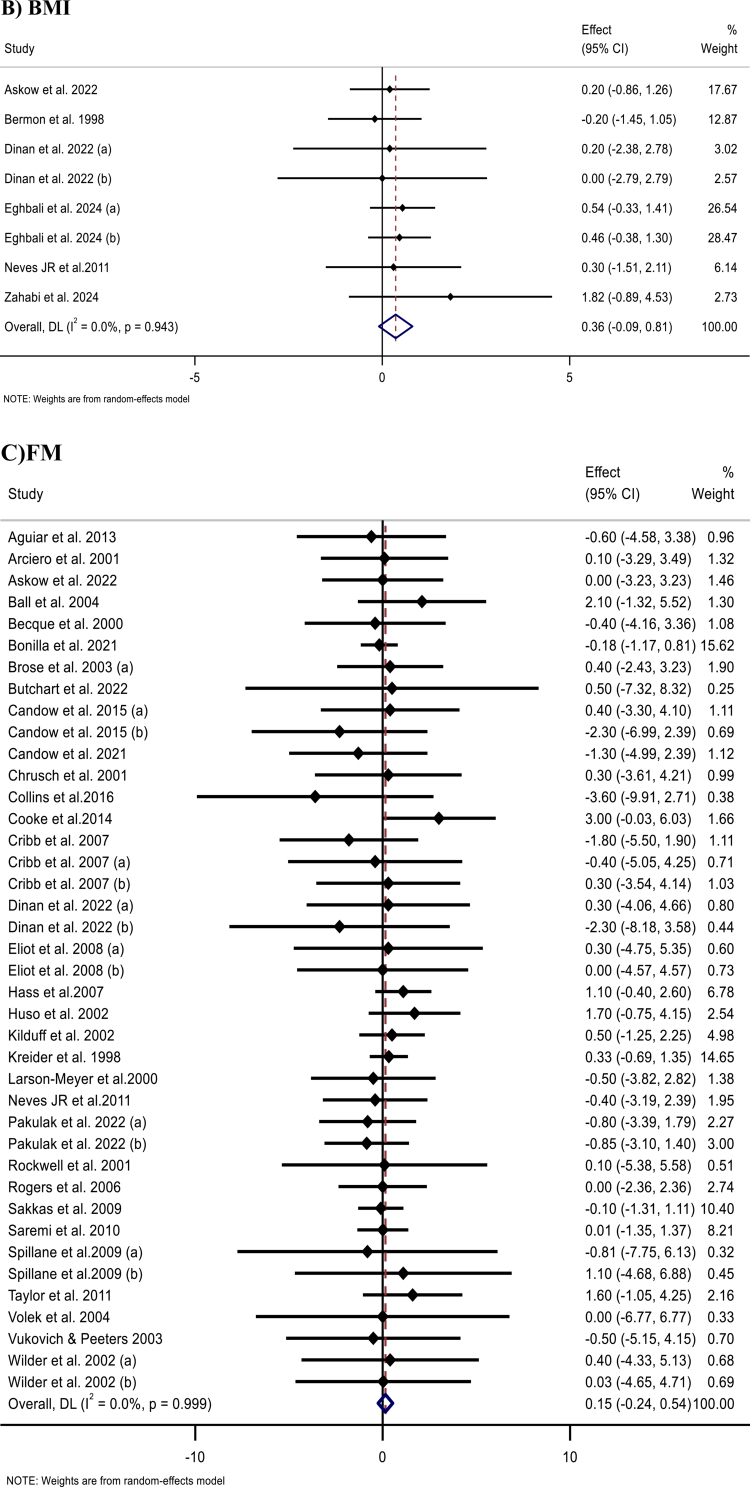

**Figure:**
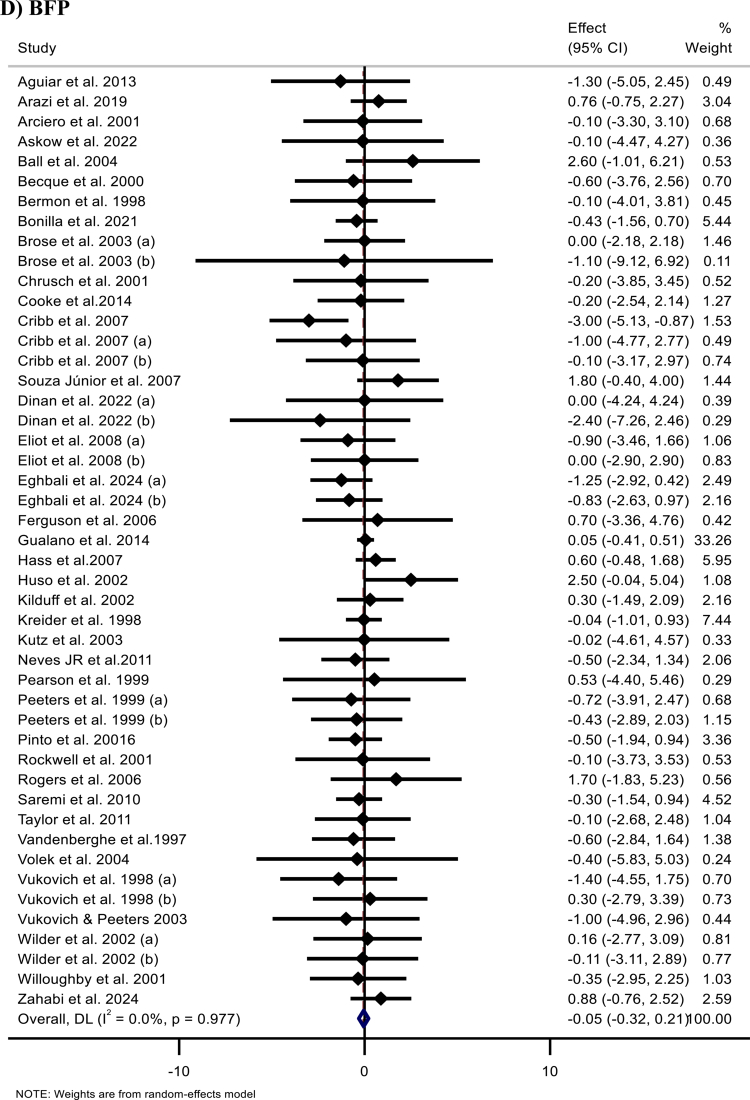

**Figure:**
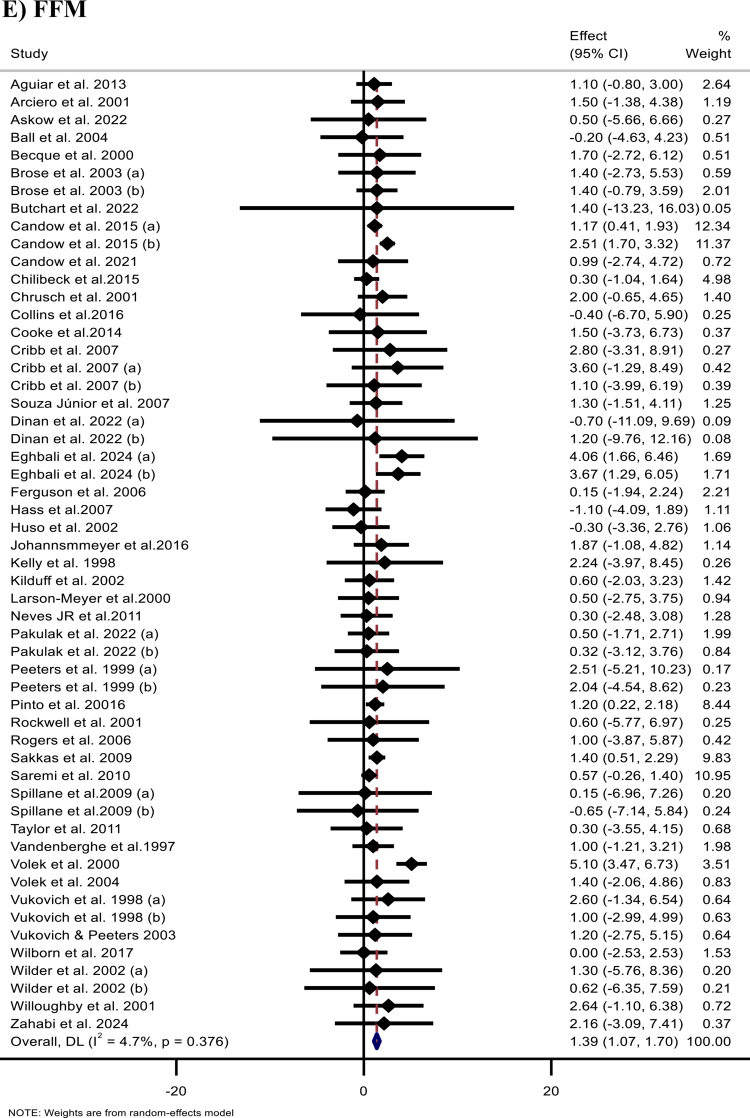

**Table 2.** Subgroup analysis of creatine supplementation effects on body composition.

Sub-groups	No. of effect sizes	WMD (95% CI)	*P-*value	Heterogeneity		
				*P-*value heterogeneity	I^2^ (%)	*P-*value between sub-groups
**Impacts of Cr supplementation on body mass** (kg)						
Overall effect	60	0.89 (0.76,1.01)	**<0.001**	1.00	0	
**Trial duration (days)**						
≤30	19	0.87 (0.75,1.00)	**<0.001**	0.999	0	0.776
>30	41	0.93 (0.56,1.30)	**<0.001**	0.998	0	
**Type of Cr**						
Cr M	57	0.87 (0.75,1.00)	**<0.001**	1.000	0	0.039
Other	3	3.59 (1.02,6.17)	**0.006**	0.831	0	
**Supplement dose (g/day)**						
≤5	17	0.93 (0.42,1.44)	**<0.001**	1.000	0	0.848
>5	43	0.88 (0.75,1.00)	**<0.001**	0.994	0	
**Total Cr dose (g)**						
≤300	27	0.88 (0.75,1.01)	**<0.001**	0.997	0	0.993
>300	33	0.88 (0.43,1.34)	**<0.001**	0.998	0	
**Baseline BMI**						
Normal	23	0.94 (0.80,1.08)	**<0.001**	0.963	0	0.062
OW	32	0.76 (0.51,1.02)	**<0.001**	1.000	0	
OB	5	−0.06 (−0.98,0.85)	0.897	0.998	0	
**Sex**						
Both	10	0.75 (−0.15,1.66)	0.105	< 0.001	0	0.896
Female	9	1.17 (−0.33,2.66)	0.126	0.913	0	
Male	41	0.88 (0.76,1.00)	**<0.001**	0.996	0	
**Age**						
≤40	45	0.88 (0.76,1.01)	**<0.001**	0.997	0	0.794
>40	15	0.77 (−0.09,1.63)	0.082	1.000	0	
**Loading**						
JL	7	0.60 (0.29,0.90)	**<0.001**	1.000	0	0.340
M	20	0.95 (0.46,1.44)	**<0.001**	1.000	0	
L + SM	7	0.93 (0.79,1.07)	**<0.001**	0.998	0	
L + LM	24	0.90 (0.34,1.47)	**0.002**	0.797	0	
HDM	2	1.45 (0.12,2.78)	**0.032**	0.938	0	
**Baseline training status**						
Trained	30	0.73 (0.49,0.98)	**<0.001**	0.983	0	0.185
Untrained	30	0.93 (0.79,1.07)	**<0.001**	1.000	0	
**Impacts of Cr supplementation on BMI** (kg/m^2^)						
Overall effect	8	0.36 (−0.09,0.81)	0.117	0.943	0	
**Trial duration (days)**						
≤30	5	0.40 (−0.10,0.90)	0.118	0.986	0	0.706
>30	3	0.19 (−0.76,1.15)	0.691	0.411	0	
**Type of Cr**						
CrM	7	0.29 (−0.23,0.81)	0.273	0.915	0	0.632
Other	1	0.54 (−0.32,1.40)	0.223	-	-	
**Supplement dose (g/day)**						
≤5	6	0.31 (−0.15,0.78)	0.184	0.952	0	0.576
>5	2	0.76 (−0.73,2.27)	0.317	0.360	0	
**Total Cr dose (g)**						
≤300	6	0.31 (−0.15,0.78)	0.184	0.952	0	0.576
>300	2	0.76 (−0.73,2.27)	0.317	0.360	0	
**Baseline**						
Normal	4	0.42 (−0.11,0.95)	0.120	0.561	0	0.662
OW	4	0.20 (−0.62,1.02)	0.629	0.999	0	
**Sex**						
Both	4	0.04 (−0.70,0.78)	0.909	0.970	0	0.554
Female	2	0.76 (−0.73,2.27)	0.317	0.360	0	
Male	2	0.49 (−0.10,1.10)	0.105	0.897	0	
**Age**						
≤40	6	0.45 (−0.04,0.94)	0.075	0.927	0	0.400
>40	2	-0.03 (−1.06,0.98)	0.942	0.655	0	
**Loading**						
M	5	0.40 (−0.10,0.90)	0.118	0.986	0	0.706
L + LM	3	0.19 (−0.76,1.15)	0.691	0.411	0	
**Baseline training status**						
Trained	5	0.52 (−0.04,1.08)	0.069	0.895	0	0.348
Untrained	3	0.07 (−0.66,0.81)	0.839	0.861	0	
**Impacts of Cr supplementation on FM** (kg)						
Overall effect	40	0.15 (−0.24,0.54)	0.462	0.999	0	
**Trial duration (days)**						
≤30	11	0.46 (−0.26,1.18)	0.210	0.979	0	0.310
>30	29	0.01 (−0.44,0.48)	0.942	0.995	0	
**Type of Cr**						
CrM	39	0.14 (−0.24,0.53)	0.476	0.999	0	0.746
Other	1	1.10 (−4.68,6.88)	0.709	-	-	
**Supplement dose (g/day)**						
≤5	12	0.10 (−0.93,1.15)	0.838	0.983	0	0.940
>5	28	0.15 (−0.26,0.57)	0.477	0.987	0	
**Total Cr dose (g)**						
≤300	15	0.56 (−0.28,1.41)	0.191	0.995	0	0.275
>300	25	0.03 (−0.40,0.47)	0.878	0.985	0	
**Baseline BMI**						
Normal	12	-0.09 (−0.65,0.46)	0.741	0.988	0	0.439
OW	23	0.30 (−0.29,0.90)	0.319	0.989	0	
OB	5	0.68 (−0.60,1.97)	0.297	0.690	0	
**Sex**						
Both	11	-0.00 (−0.87,0.87)	0.993	0.830	0	0.726
Female	3	-0.47 (−2.36,1.40)	0.620	0.997	0	
Male	26	0.22 (−0.22,0.66)	0.333	0.996	0	
**Age**						
≤40	25	0.10 (−0.36,0.58)	0.659	0.999	0	0.773
>40	15	0.23 (−0.45,0.91)	0.512	0.866	0	
**Loading**						
JL	3	0.35 (−1.21,1.92)	0.658	0.921	0	0.432
M	18	-0.26 (−0.89,0.37)	0.422	0.992	0	
L + SM	4	1.30 (−0.35,2.97)	0.124	0.817	0	
L + LM	14	0.28 (−0.36,0.93)	0.388	0.959	0	
HDM	1	0.33 (−0.68,1.34)	0.526	-	-	
**Baseline training status**						
Trained	18	-0.00 (−0.54,0.53)	0.985	0.998	0	0.426
Untrained	22	0.31 (−0.25,0.87)	0.279	0.959	0	
**Impacts of Cr supplementation on BFP** (%)						
Overall effect	47	-0.05 (−0.32,0.21)	0.701	0.977	0	
**Trial duration (days)**						
≤30	16	-0.12 (−0.71,0.45)	0.667	0.779	0	0.773
>30	31	-0.03 (−0.32,0.26)	0.832	0.955	0	
**Type of Cr**						
CrM	44	-0.04 (−0.31,0.23)	0.766	0.983	0	0.772
Other	3	-0.23 (−1.54,1.06)	0.720	0.213	35.4	
**Supplement dose (g/day)**						
≤5	18	-0.46 (−1.10,0.16)	0.149	0.999	0	0.158
>5	29	0.03 (−0.25,0.32)	0.812	0.763	0	
**Total Cr dose (g)**						
≤300	22	-0.00 (−0.58,0.57)	0.988	0.894	0	0.856
>300	25	-0.06 (−0.36,0.23)	0.671	0.907	0	
**Baseline BMI**						
Normal	17	-0.04 (−0.53,0.44)	0.862	0.607	0	0.719
OW	26	-0.10 (−0.43,0.23)	0.544	0.976	0	
OB	4	0.29 (−0.60,1.19)	0.521	0.740	0	
**Sex**						
Both	7	-0.18 (−0.60,0.96)	0.651	0.774	0	0.611
Female	7	0.04 (−0.37,0.45)	0.850	0.887	0	
Male	33	-0.18 (−0.56,0.19)	0.345	0.899	0	
**Age**						
≤40	34	-0.13 (−0.51,0.24)	0.487	0.853	0	0.551
>40	13	0.02 (−0.34,0.39)	0.889	0.985	0	
**Loading**						
JL	3	0.04 (−1.44,1.53)	0.949	0.839	0	0.135
M	18	-0.64(−1.18,-0.10)	**0.020**	0.934	0	
L + SM	6	0.79 (−0.55,2.14)	0.249	0.399	2.8	
L + LM	18	0.11 (−0.22,0.45)	0.505	0.982	0	
HDM	2	-0.03 (−0.98,0.90)	0.935	0.993	0	
**Baseline training status**						
Trained	25	-0.28 (−0.72,0.15)	0.202	0.880	0	0.192
Untrained	22	0.07 (−0.25,0.40)	0.637	0.963	0	
**Impacts of Cr supplementation on FFM** (kg)						
Overall effect	54	1.39 (1.07,1.70)	**<0.001**	0.376	47	
**Trial duration (days)**						
≤30	15	1.83 (0.89,2.76)	**<0.001**	0.747	0	0.319
>30	39	1.31 (0.93,1.69)	**<0.001**	0.218	14.5	
**Type of Cr**						
CrM	51	1.35 (1.05,1.64)	**<0.001**	0.458	0.8	0.070
Other	3	3.34 (1.21,5.47)	**0.002**	0.378	0	
**Supplement dose (g/day)**						
≤5	16	1.35 (0.73,1.98)	**<0.001**	0.686	0	0.965
>5	38	1.37 (0.95,1.80)	**<0.001**	0.208	15.3	
**Total Cr dose (g)**						
≤300	20	1.33 (0.54,2.12)	**0.001**	0.789	0	0.900
>300	34	1.39 (0.97,1.81)	**<0.001**	0.143	20.9	
**Baseline BMI**						
Normal	20	1.09 (0.64,1.54)	**<0.001**	0.629	0	0.060
OW	31	1.65 (1.19,2.10)	**<0.001**	0.339	8.1	
OB	3	-0.76 (−3.28,1.75)	0.552	0.899	0	
**Sex**						
Both	13	1.49 (1.04,1.95)	**<0.001**	0.449	4.7	0.048
Female	9	0.61 (−0.11,1.34)	0.097	0.985	0	
Male	32	1.75 (1.17,2.33)	**<0.001**	0.226	15.3	
**Age**						
≤40	37	1.47 (0.90,2.04)	**<0.001**	0.254	12.6	0.847
>40	17	1.40 (1.03,1.77)	**<0.001**	0.568	0	
**Loading**						
JL	3	0.76 (−1.30,2.83)	0.469	0.968	0	0.838
M	23	1.50 (1.11,1.90)	**<0.001**	0.449	0.8	
L + SM	7	1.05 (−0.33,2.44)	0.137	0.927	0	
L + LM	21	1.36 (0.60,2.12)	**<0.001**	0.065	34	
**Baseline training status**						
Trained	28	1.82 (1.10,2.55)	**<0.001**	0.250	14.4	0.148
Untrained	26	1.23 (0.91,1.56)	**<0.001**	0.739	0	

Abbreviations: CI, confidence interval; WMD, weighted mean difference; OW, overweight; OB, obesity; FFM, fat-free mass; BMI, body mass index; FM, fat mass; BFP, body fat percentage; L+LM, loading + long maintenance; L+SM, loading + short maintenance; HDM, high-dose maintenance; JL, just loading; M, maintenance; Cr, creatine; CrM, creatine monohydrate.

#### 3.2.2. BMI

A meta-analysis of six trials with eight effect sizes from six trials [59,72,73,90,110,111] indicated that Cr supplementation did not significantly affect BMI (WMD: 0.36 kg/m²; 95% CI: −0.09, 0.81; *p* = 0.117) (Figure 2B; Table 2). Similar results were observed in subgroup analyses.

#### 3.2.3. FM

The pooled analysis of 40 effect sizes from 33 studies [11,13,54,58–64,66–70,72,74,79,82,86,87,89,90,92,96–101,104,106,108] demonstrated that Cr supplementation had no significant effect on FM (WMD, 0.15 kg; 95% CI, −0.24, 0.54; *p* = 0.462) (Figure 2C). Subgroup analyses confirmed the robustness of these findings.

#### 3.2.4. BFP

A meta-analysis of 47 effect sizes from 39 studies [13,54,57–62,66,68–75,77,79,82,86–88,90,93–97,99,101,102,104–106,108–111] indicated that Cr supplementation did not significantly affect BFP (WMD: −0.05%; 95% CI: −0.32, 0.21; *p* = 0.701) (Figure 2D). Notably, subgroup analysis indicated that Cr supplementation loading protocols, including a maintenance phase, were associated with a modest but significant reduction in BFP, whereas other loading strategies showed no significant effect (Table 2).

#### 3.2.5. FFM

The pooled analysis of 54 effect sizes of 44 studies [11,54,58–73,75,79,82,83,85,86,89,90,92,94–110] demonstrated a significant overall increase in FFM following Cr supplementation (WMD: 1.39 kg; 95% 1.07, 1.70; *p* < 0.001) (Figure 2E). Subgroup analyses revealed significant improvements across trial durations, Cr types, dosing regimens, total Cr intake, baseline BMI categories (normal and overweight), sex (men and both sexes), age groups, loading strategies that included a maintenance phase (maintenance-only and loading plus long maintenance), and baseline training status of the participants.

### 3.3. Effects of Cr supplementation based on previous training experience

Table 3 shows the subgroup analysis of the Cr supplementation outcomes based on the participants’ previous training experience. Both trained and untrained individuals experienced significant increases in body mass (trained: + 0.73 kg; untrained: + 0.93 kg; *p* < 0.001) with a small, non-significant between-group effect (Cohen’s d = –0.36, 95% CI: –0.86, 0.15). FFM increased significantly in both trained (+1.82 kg) and untrained (+1.23 kg) participants, with a small-to-moderate, non-significant between-group effect favoring trained individuals (Cohen’s d = 0.39, 95% CI: –0.13, 0.91). Changes in BMI, FM, and BFP were minimal. These results indicated that Cr supplementation promotes FFM gain, regardless of previous training experience.

**Table 3.** Effects of creatine supplementation on body composition in trained and untrained adults.

Sub-groups	Traning status	No. of effect sizes	WMD (95%CI)	*P*-value	Between group
					Cohen’s d (95%CI)
**Body mass**	Trained	30	0.73 (0.49,0.98)	**<0.001**	−0.36 (−0.86,0.15)
	Untrained	30	0.93 (0.79,1.07)	**<0.001**	
**BMI**	Trained	5	0.52 (−0.04,1.08)	0.069	0.04 (−0.92,0.20)
	Untrained	3	0.07 (−0.66,0.81)	0.839	
**FM**	Trained	18	−0.00 (−0.54,0.53)	0.985	−0.07 (−0.60,0.45)
	Untrained	22	0.31 (−0.25,0.87)	0.279	
**BFP**	Trained	25	−0.28 (−0.72,0.15)	0.202	−0.16 (−0.74,0.42)
	Untrained	22	0.07 (−0.25,0.40)	0.637	
**FFM**	Trained	28	1.82 (1.10,2.55)	**<0.001**	0.39 (−0.13,0.91)
	Untrained	26	1.23 (0.91,1.56)	**<0.001**	

Abbreviations: CI, confidence interval; WMD, weighted mean difference.

### 3.4. Sensitivity analysis

Sensitivity analyses indicated that no single study influenced the pooled effect estimates.

### 3.5. Publication bias

Visual inspection of the funnel plots showed some asymmetry for all outcomes (Supplementary Figure 1); however, Egger’s and Begg’s tests did not provide evidence of publication bias for the outcomes.

### 3.6. Quality assessment

The overall RoB assessment indicated that 56 studies [11,54–72,74–84,86–105,107–109,111,112] were judged to have a low RoB, while five studies [60,82,91,95,112] were rated as having a high RoB (Supplementary Table 1).

### 3.7. GRADE assessment

According to the GRADE evaluation, the overall quality of evidence was high for most outcomes, including body mass, FM, BFP, and FFM. However, the quality of evidence for BMI was rated as moderate because of RoB (Supplementary Table 2).

### 3.8. Dose–response analyses

Linear and non-linear dose–response analyses (Supplementary Figures 3–5) were conducted to examine the relationships between Cr dose, supplementation duration, and body composition outcomes. In the non-linear models, Cr supplementation dose was significantly associated with changes in body mass (r = 12.90, *p* = 0.008) and BMI (r = 25.10, *p* = 0.042). Supplementation duration was substantially associated with changes in body fat percentage (BFP; r = –0.050, *p* = 0.002) and body mass (r = –0.36, *p* = 0.002). In contrast, linear dose–response models showed no significant association between Cr dose or duration and body composition changes. No significant dose–response relationships were observed for FM or FFM in either the linear or nonlinear analyses.

### 3.9. Funding and conflict-of-interest statements

Table 4 summarizes the funding sources and conflict-of-interest (COI) statements for the 61 included trials. For each study, granular entries are provided for both “Funding Source” and “Conflict of Interest Statement.” Overall, COI statements were declared in 5 studies (8.2%); 19 (31.1%) reported “none declared”; and the remaining 37 (60.7%) did not report a COI statement. Regarding funding, 37 studies (60.7%) received partial or complete industry sponsorship; 9 (14.8%) did not report funding; 3 (4.9%) explicitly reported no funding; and the remaining 12 (19.7%) were supported only by university/institutional and/or governmental sources.

**Table 4.** Funding sources and conflict-of-interest statements of included studies.

Study (author, year)	Funding source	Conflict of interest statement
[102]	Industry-sponsored + Governmental agency *Belgian National Medical Research Council (Grant G.0189.96); Novartis Nutrition (supplements); NIKE Belgium (sports outfits)*	NR
[111]	Industry-sponsored + Governmental agency *MAXIM Europe BV (provided creatine supplements); Mutualité Française, Alpes Maritimes (technical and financial support).*	NR
[87]	Industry-sponsored *(EAS grant to University of Memphis)*	Declared *(A.L. Almada cofounder/consultant for EAS)*
[112]	NR	NR
[85]	NR	NR
[105]	Industry-sponsored (*Creatine partly sponsored by Underground Sports and Fitness; Gatorade Australia supplied Gatorade*)	NR
[76]	Governmental agency + Industry-sponsored (*DG Sports of the Belgian French Community; Flamma SpA provided creatine)*	NR
[94]	Industry-sponsored (*Metabolic Nutrition Inc. and SportPharma Inc. provided creatine supplements*)	NR
[93]	NR	NR
[89]	Industry-sponsored (*Creatine monohydrate supplied by Sandco International*)	NR
[78]	NR	NR
[103]	Industry-sponsored + University/Institutional grant (*Muscular Development, Hauppauge, NY; National Strength and Conditioning Association*)	NR
[61]	NR	NR
[91]	University/Institutional grant + Governmental agency (*FWO Vlaanderen; Onderzoeksraad K.U. Leuven*)	NR
[96]	Industry-sponsored + University/Institutional grant (*NSCA and Gatorade SSI provided funding; Ross Laboratories donated formula diet; Sportpharma donated creatine*)	NR
[66]	Industry-sponsored (*MuscleTech Research and Development Inc.*)	NR
[109]	Industry-sponsored (*NutraSense, Inc.*)	NR
[80]	University/Institutional grant + Governmental agency (*K.U. Leuven, FWO Vlaanderen, Danish National Research Foundation; Technogym provided equipment*)	NR
[58]	Industry-sponsored (*Experimental and Applied Sciences, Golden, CO*)	NR
[84]	Governmental agency (*Polish State Committee of Scientific Research, grant 5 PO6K 024 10*)	NR
[46]	University/Institutional grant + Industry-sponsored (*School of Recreation and Sport Sciences, Ohio University; NutraSense Co. provided creatine*)	NR
[86]	Governmental agency + Industry-sponsored (*UK MRC; Sigma-Tau provided creatine*)	NR
[82]	Industry-sponsored (*Creatine supplied by X-Rated, Hi-Health, Scottsdale, AZ*)	NR
[88]	NR	NR
[106]	NR	NR
[62]	Industry-sponsored + University/Institutional grant + Governmental agency (*Avicena Corporation, Hamilton Health Sciences Corporation, NSERC fellowship, Canadian Foundation for Innovation*)	NR
[104]	Industry-sponsored (*Twin Laboratories provided supplements*)	NR
[60]	NR	NR
[75]	University/Institutional grant + Industry-sponsored (*University of Alberta; Allmax Nutrition provided supplements*)	NR
[97]	Industry-sponsored (*Phoenix Laboratories and B. David Tuttle supported the study; supplements from Phoenix Laboratories*)	NR
[81]	Industry-sponsored (*EAS, Inc. provided funding*)	NR
[79]	Governmental agency + University/Institutional grant (*NIH and American Parkinson Disease Association, Emory University*)	NR
[69,70]	NR	NR
[71]	Industry-sponsored (*Pro Tech Nutritional Systems of Brazil provided creatine*)	None declared
[69,70]	Industry-sponsored (*AST Sport Science supplied supplements*)	Declared (*Lead investigator was a consultant to AST Sport Science*)
[74]	NR	NR
[98]	Governmental agency + Industry-sponsored (*NIH grants; creatine and placebo donated by Jarrow Universal Herbs, Inc.*)	NR
[100]	Industry-sponsored (*Supplements donated by Labrada Nutritionals and AST Sport Science*)	None declared
[99]	NR	NR
[90]	Governmental agency + University/Institutional grant + Industry-sponsored *(Supported by FAPESP, CNPq, and Federico Foundation, supplements provided by Ethika)*.	None declared
[101]	Industry-sponsored *(Funded by Indus Biotech™; supplements packaged/administered by Indus Biotech)*	NR
[54]	Governmental agency (CAPES scholarships; CNPq grant; Araucaria Foundation partial support)	None declared
[77]	Governmental agency + University/Institutional grant + Industry-sponsored *(CNPq & FAPESP grants; support from Federico Foundation; creatine supplied by Probiotica)*	None declared
[68]	University/Institutional grant *(Baylor University Young Investigator Development Program grant)*	None declared
[65]	Governmental agency + Industry-sponsored *(Saskatchewan Health Research Foundation; Canada Foundation for Innovation; supplements from Rivalus, Inc.)*	None declared
[11]	Industry-sponsored *(Nutricia Research Foundation grant; creatine supplied by AlzChem AG)*	None declared
[95]	Governmental agency + Industry-sponsored *(FAPEG support; supplements provided by MedNutrition)*	None declared
[67]	Governmental agency + Industry-sponsored *(Fonterra & Alzchem donated supplements; author support from CNPq)*	None declared
[83]	Industry-sponsored *(Creapure® supplied by AlzChem Trostberg GmbH; no other funding reported)*	None declared
[107]	University/Institutional grant + Industry-sponsored *(Human Performance Lab, University of Mary Hardin–Baylor; product support from Dymatize Nutrition)*	None declared
[57]	Not reported (no funding source reported; CEE capsules from Labrada Nutritional, USA mentioned without financial support)	None declared
[55]	None *(no financial support for research, authorship, or publication)*	None declared
[64]	Governmental agency + Industry-sponsored *(Saskatchewan Health Research Foundation; Canada Foundation for Innovation; creatine donated by Rivalus Inc.)*	Declared *(D.G. Candow reported industry-sponsored research, donations/travel support; advisory role with AlzChem)*
[13]	Industry-sponsored *(supplements provided by MTX Corporation; APC partially funded by DBSS International SAS; conducted within DBSS “Cluster Training” project)*	Declared *(D.A.B. employed/affiliated with MTX and on AlzChem SAB; R.B.K. industry-sponsored research/support and Chair of AlzChem SAB; others no conflicts)*
[92]	NR	Declared *(D.G. Candow: industry-sponsored research, creatine donations & travel support; advisory board (Alzchem). S.C. Forbes: scientific advisor to a creatine-selling company)*
[56]	None *(no financial support for research, authorship, or publication)*	None declared
[59]	Industry-sponsored *(funded by Monster Energy Company)*	None declared
[63]	NR	NR
[72]	University/Institutional grant + Industry-sponsored *(RMUHP research grant; internal funds from Lindenwood EPNL; product support from industry)*	None declared
[73]	None (no financial support for this work)	None declared
[110]	NR	None declared

Abbreviation: NR, not reported.

## 4. Discussion

This systematic review and dose-response meta-analysis revealed significant effects of Cr supplementation combined with RT on body mass and FFM, while changes in BMI, FM, and BFP were not substantial. Subgroup analyses indicated that Cr supplementation significantly increased both body mass and FFM across various trial durations, Cr types, dosing regimens, total Cr intakes, BMI categories (normal and overweight), and baseline training status. FFM improvements were significant across all age groups in both male and mixed-sex groups, as well as loading strategies that included a maintenance phase (maintenance-only and loading plus long maintenance). Body mass increases were significant mainly in male participants and younger adults (≤40 years), as well as in all loading protocols. Cr supplementation loading protocols with a maintenance phase were associated with a modest but significant reduction in BFP. Dose–response analyses revealed non-linear associations between Cr supplementation dose and changes in body mass and BMI, with supplementation duration associated with changes in BFP and body mass. The overall certainty of evidence was rated as high for body mass, FM, BFP, and FFM, and moderate for BMI.

Subgroup analyses based on baseline training status showed that Cr supplementation combined with RT significantly increased body mass and FFM in both trained and untrained individuals, with trained participants demonstrating approximately 0.6 kg greater, but statistically non-significant, gains compared with untrained participants. Although no specific components of FFM were assessed, increases in FFM and body mass among trained individuals likely reflect muscle accretion, while in untrained individuals, they reflect water retention rather than true muscle hypertrophy [5,113]. In contrast, trained individuals may utilize Cr more efficiently for muscle protein synthesis, resulting in greater gains in actual muscle tissue. Although no studies have directly quantified the relative contributions of water and muscle mass, these physiological differences likely explain the disparities in FFM changes between trained and untrained individuals.

Cr supplementation enhances intramuscular Cr and phosphocreatine (PCr) stores, facilitating rapid adenosine triphosphate (ATP) resynthesis during high-intensity, short-duration efforts [114,115], and may indirectly promote muscle protein synthesis by increasing cellular hydration and activating anabolic signaling pathways,including the mammalian target of rapamycin (mTOR) pathway [116]. Collectively, these mechanisms contribute to muscle hypertrophy and increased FFM, with resistance-trained individuals potentially experiencing a more pronounced anabolic response than previously untrained individuals.

Although the difference in FFM increase between novice and experienced lifters was not statistically significant in the present study, the approximately 0.6 kg (≈50%) difference (1.82 vs. 1.23 kg) may be clinically meaningful for individuals engaged in resistance training [86,99], suggesting more favorable effects of Cr supplementation among those with training experience. Another important point to note is that the studies involving untrained participants had longer intervention durations. The mean duration of studies with untrained participants was 13.3 weeks,compared to 6.1 weeks in those involving trained individuals. Since changes in body composition,particularly increases in FFM,require time to develop,this difference in study duration may have influenced the findings. Previous evidence suggests that long-term Cr supplementation results in greater FFM gains over time [101]. However, despite having nearly half the intervention duration, trained individuals exhibited approximately 50% greater FFM gains than untrained participants. Given that no RCT has directly compared the effects of Cr supplementation between trained and untrained individuals under controlled conditions, such as equivalent duration, gender distribution, age, and other potential confounders, future studies are warranted to address these gaps.

One potential explanation for the greater gains in FFM observed in trained individuals is their enhanced capacity for muscle Cr uptake [117,118]. RT may enhance muscle Cr uptake [119] by improving insulin sensitivity, which facilitates Cr transport into muscle cells and enables trained athletes to achieve higher intramuscular Cr levels than untrained individuals [120]. Trained individuals exhibit a favorable anabolic environment, characterized by enhanced satellite cell activation, greater lifting capacity, and higher training volumes; additionally, a higher proportion of fast-twitch fibers contributes to greater FFM gains and increased Cr responsiveness [121–125]. Although muscle mass is only one component of FFM and changes in body water may also contribute, the combination of higher muscle Cr content and greater training stimuli explains the superior FFM gains in trained individuals compared to untrained individuals [120].

This meta-analysis indicated that Cr supplementation combined with RT produces statistically and clinically significant increases in FFM of approximately 1–2 kg [18,19,114]. These gains are meaningful for enhancing muscular strength, power output, and metabolic health, particularly in athletic populations and among individuals seeking to improve their body composition. Even modest increases in FFM have been associated with improved functional performance and reduced risk of age-related sarcopenia [126,127]. Given its well-established safety, affordability, and efficacy, Cr represents a clinically applicable and evidence-based nutritional strategy for augmenting RT adaptations in both trained and untrained individuals [14,128].

This is the first dose-response meta-analysis to examine the effects of CR supplementation combined with RT in trained versus untrained individuals. Its strengths included a large number of studies, most of which (56 out of 61) were judged to have low RoB. The GRADE assessment indicated moderate-quality evidence for most outcomes, supporting the reliability of our findings. There are several limitations, including the absence of studies that directly compare the roles of water retention and muscle growth in both trained and untrained individuals, the focus on short-term research, and the limited data on the effectiveness of Cr supplementation across various training levels. Further stuides should directly assess fluid shifts and muscle growth to clarify these differences.

## 5. Conclusion

Cr supplementation combined with RT significantly increased FFM and body mass. Although both trained and untrained individuals experienced benefits, the increase in FFM was more pronounced in trained individuals. This suggests that the body mass gain observed in untrained individuals may be due to factors other than muscle growth, such as water retention. Future studies should investigate the factors contributing to FFM gains and the mechanisms underlying variations related to training history.

## Supplementary Material

Supplementary Material Supplementary File
