# Supplementary material for: Creatine supplementation and resistance training: a comparison between novice and experienced lifters - a systematic review and dose-response meta-analysis
Source: J Int Soc Sports Nutr. 2025 Dec 23;22(Suppl 1):2586523. doi: 10.1080/15502783.2025.2586523 (PMC12777911; doi:10.1080/15502783.2025.2586523)
Supplement: Supplementary Material — Supplementary File [file RSSN_A_2586523_SM7758.docx]

**Supporting Information**

**Table S1.** Risk of bias assessment for included RCTs in the meta-analysis

| **Reference** | Bias arising from the randomization process  (Allocation bias) | Bias due to deviations from the intended interventions (Performance bias) | Bias due to missing outcome data (Attrition bias) | Bias in the measurement of the Outcome (Detection bias) | Bias in the selection of reported results (Reporting bias) | The overall risk of bias |
| --- | --- | --- | --- | --- | --- | --- |
| Aguiar et al. 2013 | L | L | L | L | L | L |
| Almeida et al. 2020 | L | L | L | L | L | L |
| Almeida et al. 2022 | L | L | L | L | L | L |
| Arazi et al. 2019 | L | L | L | L | L | L |
| Arciero et al. 2001 | L | L | L | L | L | L |
| Askow et al. 2022 | L | L | L | L | L | L |
| Ball et al. 2004 | L | L | L | L | L | L |
| Becque et al. 2000 | L | L | L | L | L | L |
| Bermon et al. 1998 | L | L | L | L | L | L |
| Bonilla et al. 2021 | U | U | L | L | U | H |
| Brose et al. 2003 | L | L | L | L | L | L |
| Butchart et al. 2022 | L | L | L | L | L | L |
| Candow et al. 2015 | L | L | L | L | L | L |
| Candow et al. 2021 | L | L | L | L | L | L |
| Chilibeck et al.2015 | L | L | L | L | L | L |
| Chrusch et al. 2001 | L | L | L | L | L | L |
| Collins et al.2016 | L | L | L | L | L | L |
| Cooke et al.2014 | L | L | L | L | L | L |
| Cribb et al. 2007 | L | L | L | L | L | L |
| Cribb et al. 2007 | L | L | L | L | L | L |
| Souza Júnior et al. 2007 | L | L | L | L | L | L |
| Dinan et al. 2022 | L | L | L | L | L | L |
| Eghbali et al. 2024 | L | U | L | L | U | H |
| Eliot et al. 2008 | L | L | L | L | L | L |
| Ferguson et al. 2006 | L | L | L | L | L | L |
| Francaux et al. 1999 | L | L | L | L | L | L |
| Gualano et al. 2014 b | L | L | L | L | L | L |
| Hamilton et al. 2000 | L | L | L | L | L | L |
| Hass et al.2007 | L | L | L | L | L | L |
| Hespel et al. 2001 | L | L | L | L | L | L |
| Hoffman et al. 2006 | L | L | L | L | L | L |

**Table S1**. Continued

| **Reference** | Bias arising from the randomization process (Allocation bias) | Bias due to deviations from the intended interventions (Performance bias) | Bias due to missing outcome data (Attrition bias) | Bias in the measurement of the Outcome (Detection bias) | Bias in the selection of reported results (Reporting bias) | The overall risk of bias |
| --- | --- | --- | --- | --- | --- | --- |
| Huso et al. 2002 | L | L | L | L | L | L |
| **Johannsmeyer** et al.2016 | L | L | L | L | L | L |
| Jówko et al. 2001 | L | L | L | L | L | L |
| Kelly et al. 1998 | U | U | L | L | U | H |
| Kilduff et al. 2002 | L | L | L | L | L | L |
| Kreider et al. 1998 | L | L | L | L | L | L |
| Kutz et al. 2003 | L | L | L | L | L | L |
| Larson-Meyer et al. 2000 | L | L | L | L | L | L |
| Maganaris et al. 1998 | L | L | L | L | L | L |
| Neves JR et al.2011 | L | L | L | L | L | L |
| Eijnde et al. 2001 | L | L | L | L | L | L |
| Pakulak et al. 2022 | L | L | L | L | L | L |
| Pearson et al. 1999 | L | L | L | L | L | L |
| Peeters et al. 1999 | L | L | L | L | L | L |
| Pinto et al. 20016 | L | L | L | L | L | L |
| Rockwell et al. 2001 | L | L | L | L | L | L |
| Rogers et al. 2006 | L | L | L | L | L | L |
| Sakkas et al. 2009 | L | L | L | L | L | L |
| Saremi et al. 2010 | L | L | L | L | L | L |
| Spillane et al.2009 | L | L | L | L | L | L |
| Taylor et al. 2011 | L | L | L | L | L | L |
| Vandenberghe et al.1997 | L | L | L | L | L | L |
| Volek et al. 2000 | L | L | L | L | L | L |
| Volek et al. 2004 | L | L | L | L | L | L |
| Vukovich et al. 1998 | L | L | L | L | L | L |
| Vukovich & Peeters 2003 | H | U | L | L | U | H |
| Wilborn et al. 2017 | L | L | L | L | L | L |
| Wilder et al. 2002 | L | L | L | L | L | L |
| Willoughby et al. 2001 | L | L | L | L | L | L |
| Zahabi et al. 2024 | U | U | L | L | U | H |

***Abbreviations:*** L, low risk of bias; H*,* high risk of bias; U*,* unclear risk of bias

**Table S2.** GRADE assessment

| **Outcomes** | **Risk of bias** | **Inconsistency** | **Indirectness** | **Imprecision** | **Publication Bias** | **Quality**  **of evidence** | |
| --- | --- | --- | --- | --- | --- | --- | --- |
| Body mass | No serious limitation | No serious limitation | No serious limitation | No serious limitation | No serious limitation | ⨁⨁⨁⨁ High |  |
| BMI | Serious limitation^*^ | No serious limitation | No serious limitation | No serious limitation | No serious limitation | ⨁⨁⨁◯ Moderate |  |
| FM | No serious limitation | No serious limitation | No serious limitation | No serious limitation | No serious limitation | ⨁⨁⨁⨁ High |  |
| BFP | No serious limitation | No serious limitation | No serious limitation | No serious limitation | No serious limitation | ⨁⨁⨁⨁ High |  |
| FFM | No serious limitation | No serious limitation | No serious limitation | No serious limitation | No serious limitation | ⨁⨁⨁⨁ High |  |

*Abbreviations:* BMI, body mass index; FFM, fat-free mass; FM, fat mass; BFP, body fat percentage

^*^It was downgraded due to the high risk of bias.

1. **Body mass**

1. **BMI**

1. **FM**

1. **BFP**

1. **FFM**

**Figure S1**. Funnel plots for the effects of Cr supplementation on **(A)** body mass, **(B)** BMI, **(C)** FM, **(D)** BFP, **(E)** FFM

1. **Body mass**

1. **BMI**

1. **FM**

1. **BFP**

1. **FFM**

**Figure S2.** Non-linear dose-response association between dose (g/day) of supplementation with Cr and absolute mean differences in **(A)** body mass (Kg), **(B)** BMI (kg/m^2^), **(C)** FM (kg), **(D)** BFP (%), **(E)** FFM (Kg). The 95% CI is depicted in the shaded parts.

1. **Body mass**

1. **BMI**

1. **FM**

1. **BFP**

1. **FFM**

**Figure S3.** Non-linear dose-response association between the duration of supplementation with Cr (days) and absolute mean differences in **(A)** body mass (Kg), **(B)** BMI (kg/m^2^), **(C)** FM (kg), **(D)** BFP (%), **(E)** FFM(Kg). The 95% CI is depicted in the shaded parts.

1. **Body mass**

1. **BMI**

1. **FM**

1. **BFP**

1. **FFM**

**Figure S4.** Linear dose-response association between dose (g/day) of Cr supplementation and absolute mean differences in **(A)** body mass (Kg), **(B)** BMI (kg/m^2^), **(C)** FM (kg), **(D)** BFP (%), **(E)** FFM (Kg).

1. **Body mass**

1. **BMI**

1. **FM**

1. **BFP**

1. **FFM**

**Figure S5.** Linear dose-response association between the duration of supplementation with Cr (days) and absolute mean differences in **(A)** body mass (Kg), **(B)** BMI (kg/m^2^), **(C)**FM (kg), **(D)** BFP (%), **(E)** FFM(Kg).
